# Gestalts at threshold could reveal Gestalts as predictions

**DOI:** 10.1038/s41598-021-97878-0

**Published:** 2021-09-15

**Authors:** Thiago Leiros Costa, Johan Wagemans

**Affiliations:** Laboratory of Experimental Psychology, KU Leuven, Leuven, Belgium

**Keywords:** Object vision, Psychology, Visual system

## Abstract

We review and revisit the predictive processing inspired “Gestalts as predictions” hypothesis. The study of Gestalt phenomena at and below threshold can help clarify the role of higher-order object selective areas and feedback connections in mid-level vision. In two psychophysical experiments assessing manipulations of contrast and configurality we showed that: (1) Gestalt phenomena are robust against saliency manipulations across the psychometric function even below threshold (with the accuracy gains and higher saliency associated with Gestalts being present even around chance performance); and (2) peak differences between Gestalt and control conditions happened around the time where responses to Gestalts are starting to saturate (mimicking the differential contrast response profile of striate vs. extra-striate visual neurons). In addition, Gestalts are associated with steeper psychometric functions in all experiments. We propose that these results reflect the differential engagement of object-selective areas in Gestalt phenomena and of information- or percept-based processing, as opposed to energy- or stimulus-based processing, more generally. In addition, the presence of nonlinearities in the psychometric functions suggest differential top-down modulation of the early visual cortex. We treat this as a proof of principle study, illustrating that classic psychophysics can help assess possible involvement of hierarchical predictive processing in Gestalt phenomena.

## Introduction

One could still argue that Gestalts are among “the best known, yet least understood, phenomena of visual perception”^1^. However, the field has come a long way since the early days where it relied mostly on phenomenology and visual demonstrations, with rare operational definitions of the phenomena and limited understanding of the neurophysiological mechanisms behind it (for reviews, see references^2,3^). We now know that some Gestalts receive a “special treatment” in the visual processing hierarchy, but we still lack a general understanding of why that would be the case and therefore miss a general theory of perceptual organization^4^.

Such special treatment could serve as a clue of how the stimuli leading to Gestalts are processed, and how and why these Gestalt phenomena emerge. Why do some combinations of items lead to a configural superiority effect (faster and more accurate detection/discrimination of a composite display than of any of its constituent parts), while most combinations of the same elements lead to the opposite effect^5^? Or, more generally, why do some feature combinations become Gestalts? One possible but still somewhat speculative answer is this: Gestalts reflect specific predictions (or *priors* in the Bayesian jargon) and are therefore used by the visual system to efficiently disambiguate visual input.

Detailing this answer to such a broad question would mean to successfully formulate a general theory of perceptual organization and is, of course, beyond the scope of the current paper. Here, we will therefore focus on a related yet more specific set of questions, easier to operationalize and tackling the same broad issue. What are the limits of “Gestaltness” and what do these limits tell us about the special nature of such phenomena? To answer these more specific questions, we developed two psychophysical studies assessing how stimuli associated with Gestalt-like phenomena are processed across the psychometric function. We will then interpret the results based on the “Gestalts as predictions” formulation^6^ and the reverse hierarchy theory^7^. Hence, the present study is offered as a sort of “proof of principle” for this view. Specifically, we will use classic psychophysical methods to argue (i) that Gestalts act somewhat like predictions in a hierarchical predictive coding framework and (ii) that parametric manipulations of the stimuli inducing these phenomena can help unveil the role of feedback between high and low visual processing areas (the reverse hierarchy part of the argument) in the processing of Gestalt phenomena*.* We present the relevant background and develop this argument further below.

### What is a Gestalt?

Vision is often considered as a hierarchical process^8^. Cells of increasing complexity, input-selectivity and response invariance have been extensively described from the retina to higher-order visual cortical areas. However, bottom-up hierarchical feature integration cannot account for many phenomena, and visual Gestalts (holistic organizations that are qualitatively different from the simple sum of their parts) are hardly ever properly explained by such an account (for a review, see^4^). When analyzed properly, these Gestalt phenomena involve complex part-whole relationships that are notably non-linear (i.e., relationships between variables that cannot be described by a constant) and often superadditive (i.e., phenomena that cannot be explained by a simple additive relationship between features). Some combinations of “parts” or features will allow for a “whole” to emerge with features of its own, that are nowhere to be found in other combinations of the same “parts”. These new emergent features will sometimes behave as the real units of perceptual processing, often being detected more accurately and faster than any of their constituent parts or basically any other related stimulus (as in the Configural Superiority Effect^9,10^).

It is also important to note that not all Gestalts are created equal when it comes to their cortical visual processing^4^. However, from what we know from neuroimaging studies so far, it seems like there is frequently a peculiar involvement of object-selective cortical areas^11–13^, often involving complex top-down modulation of the early visual cortex, especially in the Gestalt cases driven by emergence and superadditivity (i.e., a combination of elements leading to the emergence of new features associated with faster or more accurate discrimination than to any of its constituent parts shown in isolation). This top-down modulation will also often consist of inhibition of the primitive features when the Gestalt organization dominates visual awareness^14–18^. In general terms, such top-down modulation is consistent with the role of predictions in a predictive coding framework, as explained next.

### What is a prediction?

The term “prediction” can imply many things in different contexts (from fortune-telling to formal statistical models and a number of distinct phenomena in the cognitive sciences). Here, we will apply it more strictly, referring to one element of the Bayesian-inspired predictive coding models of perception^19,20^. In recent years there have been substantial new developments in our understanding of the key role that inferential mechanisms play in perception. Multiple predictive coding models have been developed and tested in an attempt to formalize how the visual system exploits the statistical regularities of the environment to save resources while making sense of the noisy sensory input (for a comprehensive review, see^21^). In brief, these models propose that perception is an active process, where higher-order areas of the brain generate hypotheses about the most likely cause of sensory input and a generative model turns these hypotheses into predictions that are fed back from higher to lower processing levels. These predictions are compared with the incoming sensory information, and the difference between these two signals is calculated in prediction error units. The main goal of this process is to maximize the accuracy of predictions with learning and action by minimizing prediction errors. This way, with basically two types of units (predictions and prediction errors) interacting in a bidirectional cascade of cortical processing (with predictions generally having a top-down influence and prediction errors acting more horizontally and bottom-up), one can explain a substantial number of phenomena in cognition and perception^22–24^. It is important to distinguish these predictions from forecasts. It is not relevant for our purposes whether these predictions refer to present or future events. Prediction here refers to the process of extrapolation from a model to new unobserved data^25^.

To illustrate this general idea, let us discuss a few specific examples. The role of prior knowledge in the disambiguation of Mooney images (thresholded black and white images that are generally perceived as abstract noise^26^) can illustrate the robustness and speed of the influence of predictions in perception. Once the observer is given information that allows for the disambiguation of the Mooney image (this may come in the form of exposition to the disambiguated image, a subtle verbal clue or even an unconsciously processed prime), the percept that was initially chaotic and ambiguous is instantly reorganized and what is depicted is recognized, a process that is also often quite persistent (e.g.,^27,28^). If seen under the light of the predictive processing models discussed above, this example shows that feeding new information into the generative model (i.e., creating a prediction) will allow for the persistent and fast disambiguation of noisy input. Specifically in the case of Mooney faces, it has been shown that such a process happens through feedback connections from the posterior fusiform face area to the occipital face area and that this activity can also be elicited by subliminal cues and expectations^29^. This rationale has also been used to assess the role of prior knowledge in the disambiguation of visual input in clinical populations (see^30^ for an example with patients with Autism Spectrum Disorder).

Visual illusions can also help illustrate the role of predictions in disambiguating sensory input through top-down modulation of the early visual cortex. For example, in a size-distance illusion (where stimulus size is under- or overestimated because of distance cues), retinotopic activation of V1 is more indicative of the perceived illusory size than of the retinal size of the stimuli^31^. Another example is the processing of modal completion in the early visual cortex, the Kanizsa triangle illusion, where the spatial context induces the perception of a white triangle occluding three black circles where there is in fact no triangle or retinal input at all. There is an increase in activity of V1 neurons across the illusory contours^32^ that seems to be driven by top-down feedback to the deeper layers of the primary visual cortex^33^. Lastly, illustrating the online and multimodal nature of these prediction-based modulations, Keller et al. have shown multiple times (e.g.,^34^) in behaving animals and at the single-cell level that cells in the visual cortex receive projection from motor areas that match expected visual input given a set of motor commands. This also serves as an illustration of possible neural correlates of prediction and prediction error units at the single-cell level.

### Gestalts as predictions

The case for Gestalts as predictions has been made elsewhere^6^. Even long before that, the likelihood principle (inspired by Helmholtz^35^) has been a guiding theoretical framework in the field, suggesting that perceptual organization will converge on a percept of the most likely source of the input. This principle is often contrasted with the simplicity principle, the idea that perceptual organization is chosen to be as simple as possible^36,37^. There have been attempts to reconcile these two opposing schools of thought under the Bayesian framework^38–42^, but not without controversy (e.g.,^43^). This controversy is beyond the scope of the current study and we will instead focus on three key observations based on empirical data that can inspire testable hypotheses.

(i) Gestalts help the visual system make sense of the noisy and unstable visual world, organizing the sensory input in patterns that have a reasonable balance between external veridicality (i.e., they allow adaptive behavior) and internal efficiency (e.g., processing time and resource allocation). This is supported by the idea that Gestalts are associated with faster reaction times, higher saliency, more efficient processing. In sum, Gestalts facilitate behavior, just like predictions do^6^.
(ii) The brain is tuned to the statistical regularities of the environment and uses the most predictive of these to disambiguate input. It has been empirically demonstrated that Gestalt principles of perceptual organization are quantitatively related to the statistics of natural images^44^. It has also been demonstrated that some principles are independent of the others and some are remarkably better predictors than others^45,46^.
(iii) In a predictive coding framework, predictions will modulate hierarchically lower areas through feedback connections and suppress activity in these areas once there is a sufficient match between prediction and sensory input. In most of the studies with methods that allow for direct assessment of multiple hierarchical levels and these feedback connections, Gestalt phenomena have been associated with differential top-down modulation of early visual processing areas that is not seen for control stimuli^18^.

Although still somewhat speculative, the Gestalts as predictions argument presented here is grounded on previous empirical work and inspires testable hypotheses. Below we will argue that in order to refine this argument we need to further assess (1) the differential involvement of higher-order areas in Gestalt phenomena and (2) the role of top-down modulation in these phenomena.

### Assessing “Gestalts as predictions” with psychophysical methods

As described above, some neuroimaging studies tend to support these hypotheses in general terms. However, we still miss systematic assessment of the notion of Gestalts as predictions with behavioral and psychophysical methods. At its best, psychophysical methods could also be regarded as “dry physiology”^47^. In fact, these tools have often unveiled key aspects of visual physiology, sometimes years before neuroimaging or other technologies were available to confirm these. Amongst these we highlight the characterization of rod and cone spectral sensitivities^48,49^ and of our light/dark adaptation dynamics^50,51^, identifying distinct spatial frequency channels subserving human spatial vision^52^, isolating our color opponent mechanisms^53^, functionally characterizing the Magnocellular and Parvocellular retino-cortical pathways^54,55^ and other critical retino-cortical specializations^56^. There are also remarkable phenomena like visual hyperacuity (e.g.,^57^), psychophysical discrimination performance that is better than one single photoreceptor receptive field. These works remind us that a thorough investigation of a physiological process can also be performed with methods that do not directly assess physiology.

There are mainly two approaches that can be used to assess the involvement of predictive processing in Gestalt phenomena. The most intuitive choice is to differentially manipulate expectations for stimuli inducing these Gestalt phenomena (e.g., stimuli leading to configural superiority; see Costa et al., in preparation). But the approach we propose here is to parametrically degrade stimuli and exploit the limits of Gestalt phenomena. For this we use classic psychophysical methods and can assess: (1) whether we still get Gestalts at and below threshold; (2) nonlinearities in Gestalt processing, and (3) the relationship between the maximal difference in performance between conditions and the presence of nonlinearities in psychometric functions**.**

The idea of parametrically degrading stimuli in order to assess hierarchical processing is inspired by the Reverse Hierarchy Theory or RHT^7,58^. In this framework, vision can be divided into two main broad processes: “vision at a glance” (initial implicit processing of stimuli generally driven by higher-order representations) followed by “vision with scrutiny” (driven by feedback connections to early visual processing areas in order to disambiguate stimuli that are somehow unclear). Degrading stimuli (i.e., presenting them at or below threshold) will increase the demands for “vision with scrutiny” and therefore differentially engage these feedback connections^58,59^. Losing Gestalt quality at threshold (i.e., no superadditivity and no difference from control conditions at threshold) would mean that there is no differential involvement of top-down modulation for the stimulus/manipulation in question. Finding sustained or even increased superadditivity and *Gestaltness* at threshold would suggest the opposite, that stimuli invoking Gestalt organizations in proper conditions (e.g., with sufficient contrast or when given enough time) can even induce top-down modulation in suboptimal conditions, confirming also that these feedback connections will play a key role in the Gestalt phenomena in questions. It is important to note that even if one does not adhere to the theoretical framework proposed here, assessing whether Gestalt quality is lost or preserved at threshold levels will inform whether the visual system has evolved to deal with these phenomena mostly in high saliency conditions. More generally, therefore, this is a question that has merit on its own.

It is also important to note that other studies have used psychophysical methods to assess different aspects of processing of Gestalt-like phenomena. For instance, this has been shown for coherent motion and shape from motion studies^60,61^, for investigations of the neural correlates of closure and figure-ground segmentation^62,63^, or in the assessment of contour integration by collinearity and local association fields^64,65^. However, psychophysical methods have not been used to assess possible involvement of predictive processing and feedback connections in the processing of emergent features when these features are isolated as proposed here (see below). This is also particularly relevant given the need to assess nonlinearities in information processing, as Gestalt theories point to emergence as a nonlinear process.

The manipulation of stimulus parameters across a psychometric function (i.e., from levels of low accuracy to levels where performance saturates) can also unveil nonlinearities in the processing of stimuli inducing Gestalt phenomena. We argue here that this can also help diagnose the differential involvement of higher-order visual processing areas in these phenomena (and ultimately, that the assessment of the relationship between nonlinearities and the abovementioned strengthening or weakening of Gestaltness at threshold can unveil key insights about the predictive processing behind these phenomena). In fact, emergence (the process through which new properties appear when stimulus elements are combined into a unitary configuration) is at the core of what defines a Gestalt and is by definition non-linear (as it is non-additive; e.g.,^5^).

The steepness of psychometric functions has reliably been used as an indicator of the differential involvement of high and low processing levels in visual perception (for a review of the argument focusing mostly on contrast responses, see^66^). This is illustrated in a magnetoencephalography study by Hall et al. (2005). The authors found that contrast response functions derived from the striate cortex were somewhat linear, while the extra-striate cortex had a steeper and non-linear function. This supports the well-established notion that, along the hierarchy of cortical visual processing, responses change from being dependent on stimulus energy (e.g., contrast, size) at low levels to being percept and information-dependent at higher-order areas (for other examples, see^67–69^. In the field of pure psychophysics, Brown et al.^70^ found that the changes in illusion magnitude as a function of stimulus contrast were linear for illusions that relied more strongly on low processing levels (e.g., simultaneous brightness and simultaneous tilt) and nonlinear for illusions that rely more strongly on contextual information and higher processing levels (e.g., the Ponzo and Poggendorff illusions).

### A proof of concept study

Above we have made a case for why modulating stimuli associated with Gestalt phenomena across the psychometric function can help assess the hypothesis of “Gestalts as predictions”. We now propose a sort of proof of principle investigation to fill a few critical gaps in this literature and inspire more nuanced investigations of this subject in the future. We will use two complementary stimulus sets and tasks.

In Experiment 1, the classic line stimuli inducing the configural superiority effect (CSE) and configural inferiority^9^ will be used in a four alternative forced-choice task. These are archetypical examples of Gestalt phenomena and have also been investigated with multivariate pattern analysis in an fMRI study, showing that the Gestalt phenomena emerge mainly at the LOC and that the control stimuli engage mainly the early visual cortex (^11^; with support from an EEG study;^71^). In this experiment we will have one condition where we manipulate the contrast of the stimulus and another condition where a mask with luminance noise will be added on top of the contrast manipulation. This will allow for a more extensive assessment of the RHT-inspired hypothesis that degradation of stimuli will differentially engage feedback mechanisms for Gestalts (i.e., how does this further degradation of viewing conditions affect the shape of the psychometric function and the distribution of the differences between Gestalt and control conditions across contrast levels?).

Although well-accepted in the field, the line stimuli used in Experiment 1 have some limitations. One could consider familiarity as a possible confounder of classic CSE studies since triangles and arrows are well known shapes and familiarity is known to play a role in figure-ground segregation^72,73^. Even though extensive data is present in the literature suggesting CSE is induced in a similar way with different tasks and unfamiliar stimuli (see^5^ for a review or^13^ for an example with converging neuroimaging and behavioral results), familiarity could still play some role. Therefore, it is important to assess our hypotheses with other stimuli and tasks (see below).

Another potential caveat is that these are comprised of multiple possible Gestalt-like features and it is hard to attribute the increased saliency and the superadditivity to any of these (for a detailed argument, see^10^). There are also multiple interactions between line elements (parallelism, T-junctions, etc.) that can confound visual discrimination^74^. Inspired by some of these issues and the need to isolate specific grouping principles and emergent features, different authors have tried to devise some “pure Gestalts” using unconnected dot elements^10,75,76^. These authors have found that a pair of dots presented in a change detection task can show superadditive Gestalt-like characteristics and that different grouping principles can be isolated with this approach. Two isolated unconnected dots will be processed as one single object when they simultaneously move in space, and there are differences in performance and information processing mechanisms when this emerging percept simply changes location or when these changes lead to the emergent features of orientation or proximity^75^, replicated in^76^. These information processing peculiarities are coherent with Gestalt theories of perceptual organization (in contrast to local theories of feature integration) and have been shown to be specific to the different emergent features and not reflecting a general processing gain behind feature changes^76^.

Experiment 2 uses this dot task to assess whether any effects found in Experiment 1 are also present when specific grouping principles are isolated, when a different task is used and when familiarity is controlled for. This time, instead of contrast, we will use stimulus displacement as the independent variable manipulated across the psychometric function. Using luminance contrast on Experiment 1 and local displacement in Experiment 2 (therefore engaging clearly different low-level mechanisms) will also allow us to assess how much the effects of saliency manipulation of Gestalts can be extrapolated across different visual processing mechanisms.

## Methods and results

### Experiment 1

Experiment 1 consists of the reanalysis of data from two experiments recently reported elsewhere by the authors^77^. Upon the conception of the study that would lead to the present manuscript and the previously mentioned one, the authors had two goals. One of these was to replicate and extend the study by Bratch et al.^78^, looking at clarifying the claims of less efficient information processing and higher thresholds for CSE-inducing stimuli when compared to their constituent parts (a claim that is in conflict with the previous literature on the matter, see^77^). Another key goal we had was to properly assess how contrast and saliency manipulations could help us understand the processing of configural Gestalts and potentially unveil a differential role of predictive processing for these stimuli (one of the main goals of the current paper). We agreed that these two goals would not coexist well in one single paper (given the focus on replication and extension of the claims by Bratch et al.^78^, in Moors et al.^77^) and that a separate analysis of the relevant parts of the data, combined with new experiments was the best way forward to achieve the second goal. We now analyzed the data to include only the conditions with the same number of elements and similar spatial frequency content (excluding some conditions that were relevant only for a comparison with the work of Bratch et al. and not relevant to address our current questions). We also adopted a simpler modeling of the data to assess at which data point (from a sample of actually recorded data points) discrimination the Gestalt conditions differed from the control conditions and how that relates to the threshold. We refer to Moors et al.^77^, for more detailed information.

#### Participants

We tested 20 participants with normal or corrected-to-normal visual acuity (assessed before the first testing session). The experiment was divided in two sessions of approximately 1 h each performed on separate days (one for stimuli with noise and another for noiseless stimuli). The order of the sessions was counterbalanced across participants. Participants received course credit or financial compensation and provided written informed consent according to international standards (approved by the KU Leuven Social-Societal Ethics Committee). All procedures were performed in accordance with the declaration of Helsinki.

#### Equipment and procedure

Stimuli were presented on a linearized CRT monitor (Sony GMD F520) with a refresh rate of 60 Hz. Stimuli were presented against a gray background with and average luminance of 38 cd/m^2^ using the Bits# system (Cambridge Research Systems) to allow for a 14-bit contrast resolution. Experiment parameters were controlled by a software written in Python 2.7 relying on the PsychoPy library^79^. Subjects kept a viewing distance of 57 cm with the help of a chinrest.

**Figure 1:**
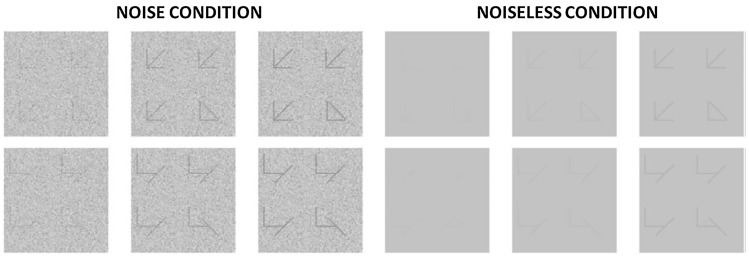
Examples of three levels of contrast manipulations for CSE (top) and Control stimuli (bottom) in the noise and noiseless conditions. In this example, contrast increases from the leftmost to the rightmost display. Adapted from Moors et al.^77^.

Each stimulus consisted of a display with 4 quadrants of 80 × 80 pixels, each of which contained one target. Each display was presented in the center of the screen having approximately 4 × 4 degrees of visual angle. For the CSE condition, the targets were three arrows and one triangle (see Fig. 1). All targets were drawn with the same three line elements, but the organization of these elements differed between targets. For the control condition, the targets consisted of different organizations of the same three line elements used in the CSE condition. A key difference between these two conditions is the absence of the emergent features of closure and “pointing” leading to Gestalt-like phenomena in the control conditions (for an assessment of the rationale behind the use of these displays and the diagnostics of these emergent features, see^5,9^). In every display, three items are the same and one is different from the others. The location of the odd quadrant varied across displays (with a 25% probability of being present in one of the quadrants). The task of the participant was to identify which of the four quadrants had the odd item (i.e., the target) by pressing one of four keys on the numeric keyboard (“4”, “5”, “1” or “2”; with “4” for the upper left quadrant and the other numbers for their respective positions). Stimuli were presented for 300 ms, preceded by a 500 ms fixation point. Participants were instructed to be as fast and accurate as possible.

Here, we excluded the “part” condition reported in Moors et al.^77^, as these consisted of one single line per quadrant. If we had kept such a condition, we would be comparing stimuli comprised of a different number of elements and spatial frequency content, therefore being differentially affected by contrast manipulations. On top of this, single-line stimuli such as these can induce “false pop-out”, an anti-metamere phenomenon that can also induce configural processing and be a remarkable confounder here (see^80^). One control condition is sufficient given the purposes of the current study, and the one we chose to use here is clearly more adequate (i.e., exact same local elements but distinct global configuration).

Contrast of the pixels (c_*i*_) at pixel location *i* was defined as: C_*i*_ = (L_*i*_ − L)/L, where L_*i*_ is the luminance of the pixel in question and L is the average luminance. For the noise condition, homogenous external noise was added after the contrast manipulation mentioned above (a random selection of pixel intensities derived from a Gaussian distribution with a variance of 0.062).

We used the psychophysical method of constant stimuli, with 7 contrast levels for each condition, spaced logarithmically. The range of contrast values used was different for the noise (0.1 to 0.3) and noiseless (0.01 to 0.08) conditions and was determined based on pilot experiments in order to ensure that the lowest values were at chance performance and that performance would not saturate too early, resulting in a typically sigmoidal psychometric function shape. There were 60 trials for each combination of stimulus type and contrast, resulting in 840 trials in each session and a total of 1680 trials per participant in total.

#### Analyses

Data was analyzed using R 3.5.1 in Rstudio^81^, relying on Quickpsy^82^ and suggestions from Knoblauch and Maloney^83^. Individual psychometric functions (incorporating guess and lapse rates according to suggestions by Wichmann and Hill^84^) were calculated for each participant and each condition. Thresholds for each condition were set at 60% proportion correct responses (see^77^ for the rationale on this threshold choice). For each condition we performed a repeated-measures ANOVA with two within-subject factors: stimulus and contrast. This was performed for both accuracy and reaction time (for correctly responded trials only) data. Post-hoc pairwise comparisons were performed with false discovery rate correction for multiple comparisons.

#### Results

**Figure 2:**
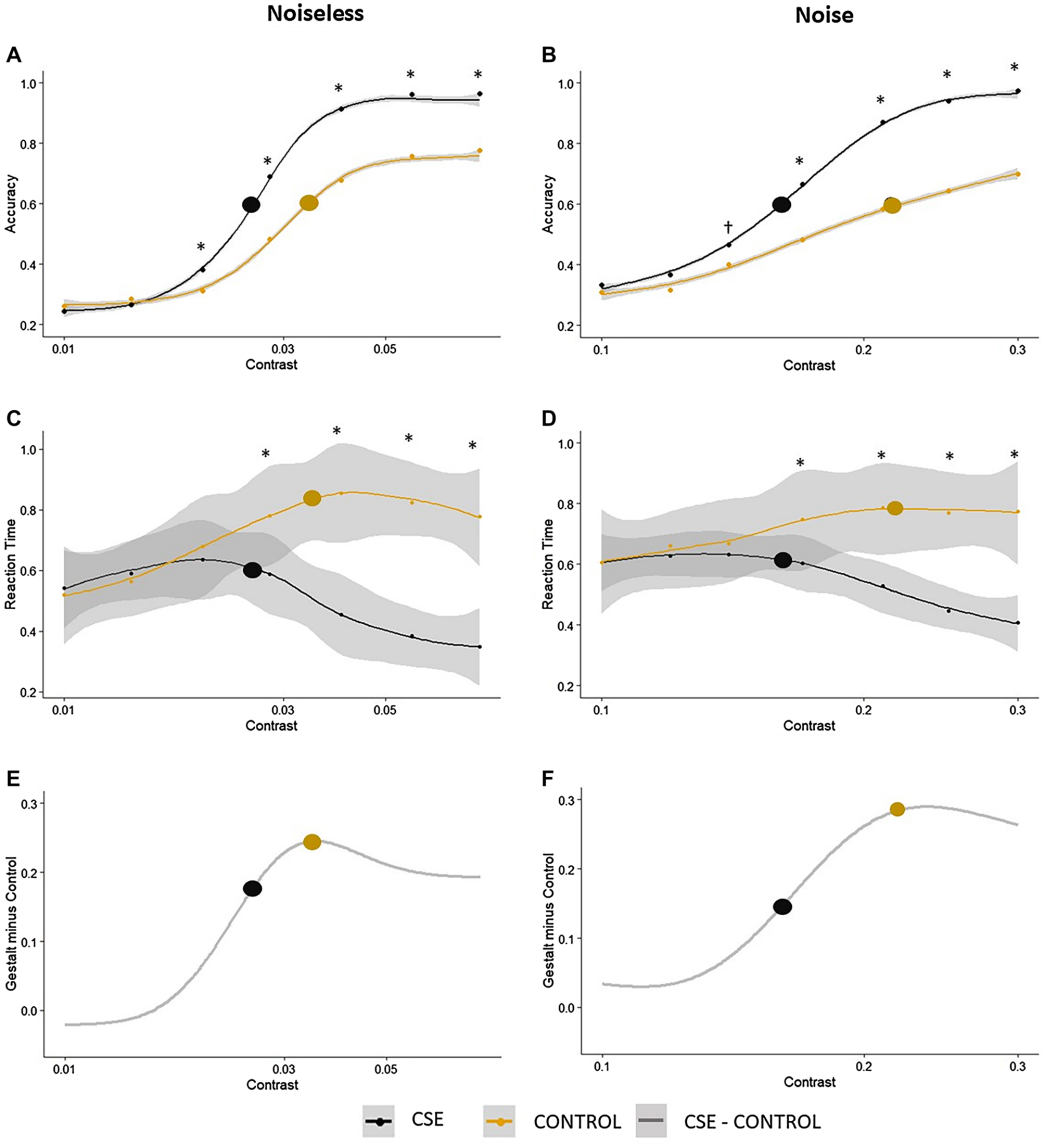
Results for Experiment 1. (**A**) and (**B**) represent the aggregate psychometric functions for the noiseless and noise conditions, respectively (locally estimated scatterplot smoothing; i.e., LOESS fit). Panels (**C**) and (**D**) represent the average reaction time data for the noiseless and noise conditions, respectively (LOESS fit). Small dots represent the average response for each contrast level and large circles represent the average threshold for each condition. Asterisks indicate the data points at which significant differences were found. The cross symbol in panel (**B**) represents borderline statistical significance (*p* = 0.06). The shaded gray area represents the standard errors of the mean. Panels (**E**) and (**F**) show the difference between the psychometric functions for the Gestalt and Control conditions, for the noiseless and noise conditions, respectively.

There was a significant effect for the factors stimulus and contrast and a significant interaction between factors for accuracies and reaction times in both conditions (noise and noiseless). ANOVA results are summarized in Tables 1 and 2. For the accuracy data, post-hoc pairwise comparisons showed that CSE stimuli differed from control at all contrast levels from the third lowest contrast upwards for the noiseless condition (from 0.02 to 0.08, *p* < 0.01) and from the fourth lowest contrast upwards for the noise conditions (from 0.17 to 0.3, *p* < 0.01). For the noise conditions there was also a borderline significant effect for the third lowest contrast level (*p* = 0.060). See Fig. 2 for more details. For the reaction time data, post-hoc pairwise comparisons also showed that CSE stimuli differed from control at all contrast levels from the fourth lowest contrast upwards for both the noiseless (from 0.028 to 0.08, *p* < 0.01) and noise conditions (from 0.17 to 0.3, *p* < 0.01).

**Table 1:** Summary of ANOVA results (accuracy).

	Df (residuals)	F	*p*	ƞ_p_^2^
**Noiseless condition**				
Stimulus	1 (19)	47.84	< 0.001	0.91
Contrast	6 (119)	209.9	< 0.001	0.71
Stimulus × Contrast	6 (119)	20.98	< 0.001	0.52
**Noise condition**				
Stimulus	1 (19)	53.54	< 0.001	0.92
Contrast	6 (119)	226.4	< 0.001	0.73
Stimulus × Contrast	6 (119)	21.45	< 0.001	0.53

**Table 2:** Summary of ANOVA results (reaction times).

	Df (residuals)	F	*p*	ƞ_p_^2^
**Noiseless condition**				
Stimulus	1 (19)	41.82	< 0.001	0.68
Contrast	6 (114)	6.89	< 0.001	0.26
Stimulus × Contrast	6 (114)	36.28	< 0.001	0.65
**Noise condition**				
Stimulus	1 (19)	46.82	< 0.001	0.71
Contrast	6 (114)	4.13	< 0.001	0.17
Stimulus × Contrast	6 (114)	21.83	< 0.001	0.53

Average thresholds were 0.025 and 0.034 for CSE and control, respectively, in the noiseless condition and 0.16 and 0.21 in the added noise condition. This makes it clear that we found significant differences between stimuli way below threshold performance for both reaction times and accuracies. In fact, stimuli start differing from each other slightly above change performance (at contrast levels 0.02 and 0.14 in the noiseless and noise conditions, respectively, see Fig. 2A, B).

We found that the maximal difference between stimuli for both noise (Fig. 2B) and noiseless (Fig. 2A) conditions is found around the threshold for the control conditions; Gestalt thresholds happen way sooner, being apparently a better predictor of differences in reaction times between conditions (see Fig. 2C, D). Also, peak differences between Gestalt and control conditions seem to happen around the time where responses to the Gestalt stimuli start to saturate. This mimics somehow the behavior of extra-striate and striate cortex neurons when contrast manipulations are performed^85^.

### Experiment 2

Task and stimuli used here are adapted from the work of Hawkins et al.^75^ given their goal to minimize confounders and extend the proposal of Pomerantz and Portillo^11^ to use dots to compare emergent features such as orientation and proximity on a common scale (as discussed in the “Introduction” section). Here, instead of contrast, we now parametrically manipulate stimulus displacement in a change detection task.

#### Participants

For Experiment 2 we tested 14 participants with normal or corrected-to-normal visual acuity (assessed before the first testing session). From these, one participant had to be excluded after giving random responses to all stimuli (leading to chance or near-chance performance across all the different stimulus levels), leaving us with 13 participants. Participants were unaware of the specific purposes of the study, received course credit or financial compensation and provided written informed consent according to international standards (approved by the KU Leuven Social-Societal Ethics Committee). All procedures were performed in accordance with the declaration of Helsinki.

#### Equipment and procedure

**Figure 3:**
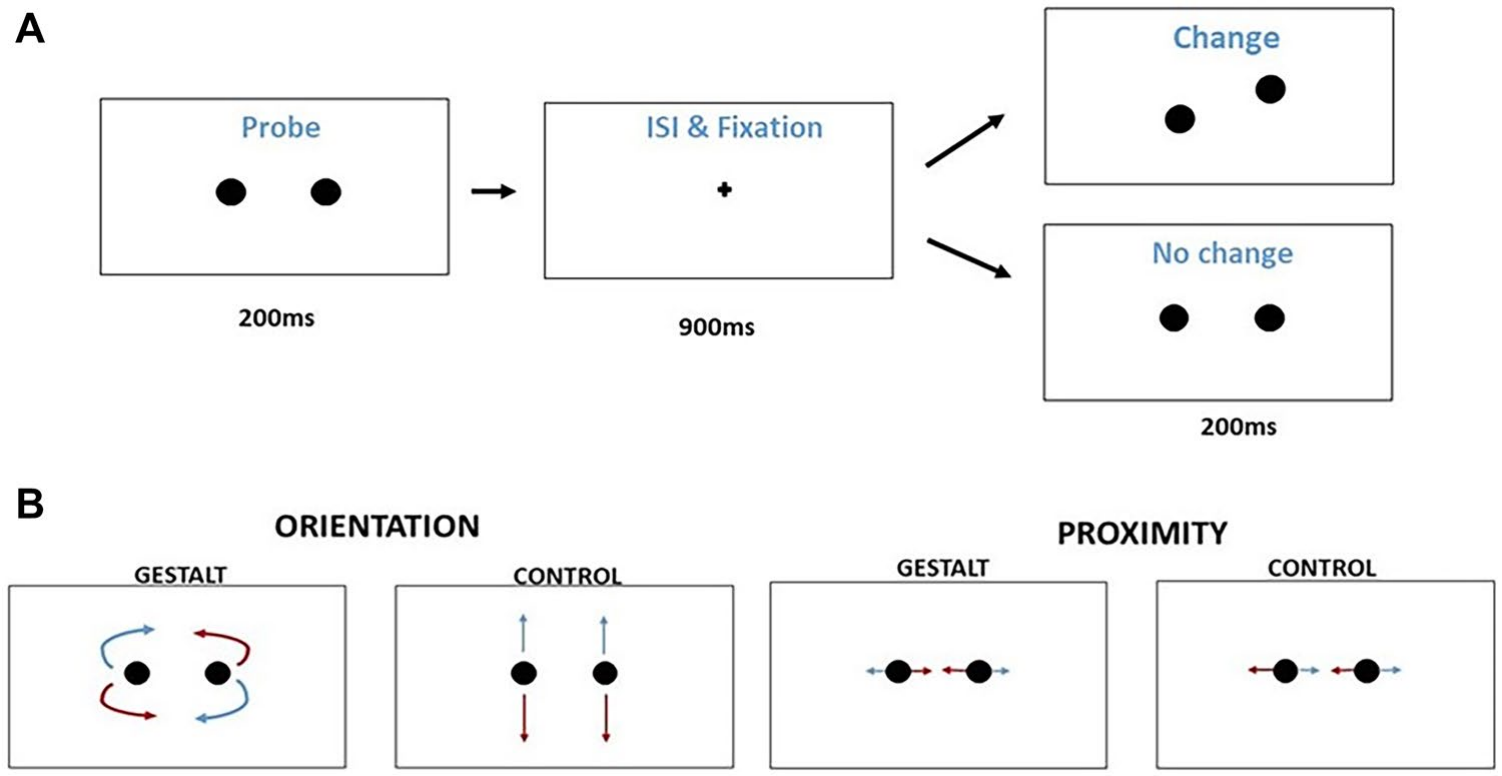
Schematic representation of the stimulus presentation sequence within a trial (**A**) and of the different changes in dot location that created the different stimulus conditions (**B**). (**A**) Each trial starts with the presentation of a reference stimulus, followed by a fixation dot and the target stimulus (that could be the same reference stimulus or one where there was a change in location of the dots). The task of the participant was to report whether there was a change in location of the dots or not. (**B**) Examples of the 4 possible types of stimulus changes. Changes for the Gestalt conditions led to a change in the configuration of the percept: dots getting closer or more distant from each other (proximity) or dots moving around a circular axis without changes in proximity (orientation). For the control conditions, dots would only change location without changing configuration (moving coherently up or down for orientation control or left and right for proximity control). For more information on the rationale behind the development of these stimuli and the use of a change detection task, see^67^.

For this experiment, we used the same parameters and equipment as Experiment 1. However, as no manipulations of contrast were performed here and stimulus rendering demands were straightforward, the Bits# device was not used. The task consists of change detection where stimuli are two black dots that change in location leading to a change in grouping demands (Gestalt conditions) or a simple change in location with no change in grouping demands (control conditions). There is an equal probability of change or no change in each trial (i.e., chance performance is at 50% accuracy). The two black dots are located 160 pixels from each other and equidistant from the center of the screen by 80 pixels. Stimuli were observed from a distance of 57 cm from the screen, and the global percept of the 2 black dots comprised 4 degrees of visual angle in size in all conditions except when proximity was manipulated (see below). Each trial consists of a sequence of three events: (1) a pair of dots is presented for 200 ms; (2) an interstimulus interval of 900 ms, with a fixation point in the center of the screen; and (3) the target stimulus is presented for 200 ms. Responses are followed by a 300 ms inter-trial interval (Fig. 3).

The target consisted of the same two dots presented in the beginning of the trial and the dots could have changed location or not. These changes in location could happen in one of 4 qualitatively different ways. (1) Gestalt conditions for proximity: The dots could get closer or more distant from each other, without any other changes in location or orientation of the global percept. (2) Gestalt conditions for orientation: The dots remain at a fixed distance but change location at an axis around the center of the screen. This way the global percept changes orientation clockwise and counterclockwise without changes in proximity of the local elements. (3) Control conditions for proximity: The dots do not change their proximity from each other and move simultaneously towards the left or right of the screen across the horizontal plane. (4) Control conditions for orientation: The dots do not change their proximity from each other and move simultaneously up or down in the vertical plane (control condition for orientation).

This way, the stimuli in the Gestalt condition and their respective control conditions fall within almost identical visual field locations and are built using exactly the same elements. The only difference between stimuli are the emergent features generated by the changes in position of the dots. The Gestalt conditions have changes in the emergent features of orientation and proximity after the location change, while the control conditions only change the location of the percept.

We used the psychophysical method of constant stimuli, with six levels of displacement of the dots (2, 5, 10, 20, 30 and 40 pixels from the initial location of the dots). These displacement values were based on pilot studies. Also, the displacement value of 2 pixels seemed like conservatively the smallest value we could reliably generate. We chose values where we had a reasonable number of data points in the rising phase of the psychometric function for all stimulus types (avoiding too many data points after responses saturate). As we are dealing with a two-alternative forced-choice task, we set thresholds at 82% correct (therefore making it equivalent to a 3-down 1-up staircase rule in a standard staircase procedure). In total we had 1920 trials, with half of these being “no change” trials. For the change trials, we had 40 trials per each stimulus and displacement level combination.

#### Analyses

Data was analyzed with the same rationale as in Experiment 1: a repeated-measures ANOVA with 2 within-subject factors: stimulus type (with 4 levels) and displacement magnitude (with 6 levels). This was done for both accuracy and reaction time (for correct responses only) data. Post-hoc pairwise comparisons were performed with false discovery rate correction for multiple comparisons.

#### Results

Table 3 summarizes the ANOVA results for both accuracy and reaction time data. We found significant effects of the factors displacement magnitude and stimulus type as well as a significant interaction for both accuracy and RT analyses. Participants were more accurate and faster to detect changes in the Gestalt conditions. Post-hoc pairwise comparisons for the accuracy data showed that Orientation differed from control for the displacement values from 2 to 20 pixels (*p* < 0.01) and that proximity differed from control from 10 to 40 pixel displacement values (*p* < 0.01). Also, the Gestalt conditions of orientation and proximity differed from each other for all displacement values from 2 to 20 pixels (*p* < 0.01).

**Table 3:** Summary of ANOVA results.

	Df (residuals)	F	*p*	ƞ_p_^2^
**Accuracy**				
Stimulus	3 (36)	110.8	< 0.001	0.9
Displacement	5 (60)	244.1	< 0.001	0.95
Stimulus × Displacement	15 (180)	22.13	< 0.001	0.64
**Reaction time**				
Stimulus	3 (31)	18.94	< 0.001	0.64
Displacement	5 (55)	19.16	< 0.001	0.63
Stimulus × Displacement	15 (171)	2.2	< 0.001	0.16

For the reaction times, however, orientation was significantly different from control conditions for the stimuli with 10 pixels of displacement only (*p* < 0.01). Proximity was only different from controls at 30 pixels of displacement (*p* = 0.05). For the comparisons between Gestalt conditions, orientation differed from proximity for all displacement levels from 2 to 20 pixels (*p* < 0.05).

**Figure 4:**
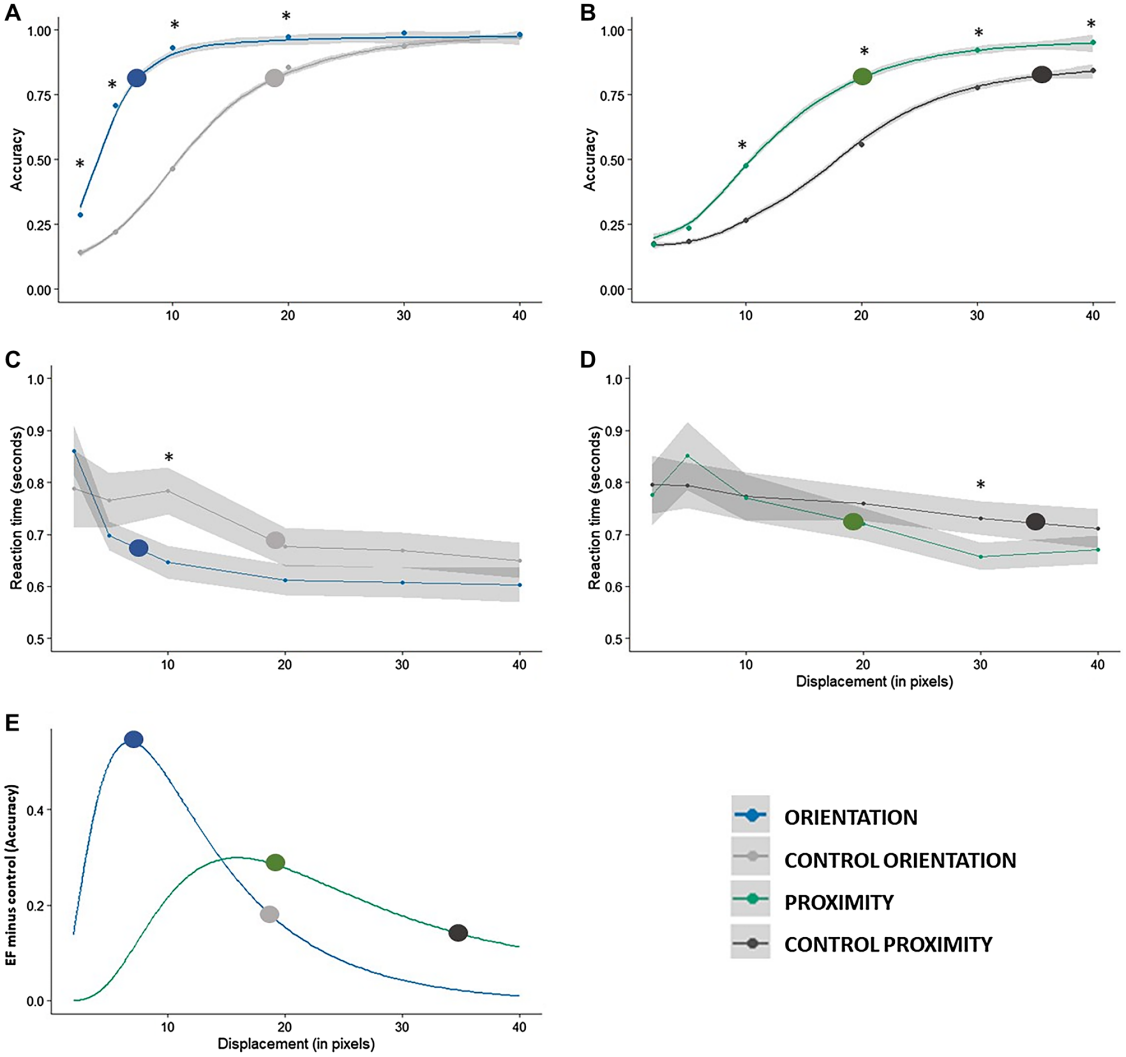
Results for Experiment 2. (**A**) and (**B**) represent the aggregate psychometric functions for the orientation and proximity conditions, respectively (LOESS fit). Panels (**C**) and (**D**) represent the aggregate reaction time data for the orientation and proximity conditions, respectively. Small dots represent the actual data points measured and large circles represent the average threshold for each condition. Asterisks indicate the data points at which significant differences were found. The shaded gray area represents the standard errors of the mean. Panel (**E**) shows the difference between the aggregate psychometric functions of Gestalt and control conditions for both orientation and proximity.

Average thresholds were 6.55 and 20.02 pixels for orientation and proximity, respectively, and 18.88 and 35.53 for their respective control conditions. Taken together, these results suggest that, as in Experiment 1, Gestalt-like stimuli were significantly different from the respective control conditions way below threshold level (and even at chance performance for both orientation and proximity, see Fig. 4A, B). Here, similarly to Experiment 1, peak differences between Gestalt and control conditions happened around the time the Gestalt responses were starting to saturate, and this time, remarkably close to the Gestalt threshold (see Fig. 4E). For this experiment, though, reaction time data was somewhat less informative. This can in part be explained by the different methods (a four alternative forced-choice approach in Experiment 1 and a change detection method here).

## Discussion

We used two tasks and two different kinds of Gestalts to assess how these phenomena behave across the psychometric function (i.e., from chance to ceiling performance). In Experiment 1 we employed contrast manipulations (and added luminance noise) and in Experiment 2 we had manipulations of configurality based on the physical displacement of stimulus elements in a change detection task. Our main goal was to assess whether there is differential involvement of higher-order object-selective areas in these phenomena (diagnosed here through nonlinearities in psychometric functions; e.g.,^85^) and whether there is peculiar top-down processing involved (diagnosed here through the robustness of Gestalt phenomena against saliency manipulations, an argument inspired by RHT, see “Introduction” section) as a way to investigate whether there is differential predictive processing of visual Gestalts. Our results clearly support this hypothesis.

The current approach can also be considered an extension of the proposal of Pomerantz and Portillo^11^ for diagnostics and comparison of visual Gestalts: (1) define them operationally via emergent features, and (2) quantify *Gestaltness* using performance on standard discrimination tasks, so that different grouping and superadditive phenomena can be compared on a single scale of measurement (e.g., discrimination accuracy or reaction time). Differences in accuracy of discrimination performance are straightforward and adequate tools to diagnose Gestalts (see^11^ for an extensive articulation of this point). Both experiments reported here show that this *Gestaltness* is maintained across the full psychometric function, with a robust configural superiority effect (i.e., significant differences between discrimination of stimuli containing emergent features and stimuli comprised of their constituent parts in isolation or organized differently) as soon as we observe above-chance performance (i.e., at the first data point where performance is above 25% correct in Experiment 1—see Fig. 2A—and around 50% correct performance for both orientation and proximity emergent features—see Fig. 4A, B).

Gestalts are percepts and not stimuli. Therefore, it is not straightforward to talk about the emergence of Gestalt-like phenomena when stimuli are degraded to the point where discrimination performance is below threshold and just above chance. In such conditions it is safe to propose that there is also limited awareness of stimulus features and one could argue that speaking of Gestalt qualities in such conditions is a long stretch. However, the idea that there is no proper dichotomy between early pre-attentive and late attentive processes in perceptual organization, with "gradual emergence of *Gestalten*" (referred to as "microgenesis" in Gestalt psychology) has long been a viable hypothesis (e.g.,^4^). This idea has found empirical support in the work of Kimchi^86,87^ with primed matching paradigms and, less specifically, in recent studies assessing neurophysiology^13,18,86,87^. The data presented here further supports this notion.

Beyond accuracy differences for stimuli presented below threshold, conditions inducing Gestalt phenomena were also associated with remarkably steeper slopes of the psychometric functions for all stimuli in both experiments (Figs. 2 and 4). For Experiment 1, slopes become shallower once luminance noise is added, but *Gestaltness* remains unaffected or even stronger when compared to noiseless conditions (see slightly larger effect sizes for accuracies—Table 1—and larger differences between Gestalt and control conditions when noise is added—Fig. 2E, F). This suggests that the more we degrade the stimuli in question (which in the logic of RHT also means to engage stronger top-down modulations), the stronger the Gestalt effect.

It is worth noting that for both experiments, peak *Gestaltness* (i.e., peak differences between Gestalt and control conditions) was found at the end of the rising phase of the psychometric function and around the time where responses to Gestalt conditions were starting to saturate (i.e., reach ceiling performance, see Figs. 1E, F and 2E). This is remarkably similar to what was described in Hall et al.^85^ when comparing contrast response functions (CRFs) derived from striate and extra-striate cortex with magnetoencephalography. Peak differences between striate and extra-striate CRFs were found around the time where striate cortex responses start to saturate (for more comprehensive figures, see also Brown et al., 2018). In this context, one can interpret the saturation of extra-striate activity in the CRF as the peak of information-driven/percept-driven processing (as opposed to energy-driven/stimulus-driven processing; e.g.,^67,70^). Here we propose to extrapolate over the methodological differences between our study and Hall et al.^85^, and to interpret this result as indicating that (1) peak *Gestaltness* is observed when the visual system switches from stimulus- and energy-dependent processing to percept- and information-dependent processing and that (2) Gestalts differentially engage higher-order visual processing areas, even when dealing with “pure Gestalt” (i.e., the two-dot pure emergent feature stimuli). The fact that the same behavior of the psychometric function was found in both experiments and therefore can be extrapolated from contrast processing (Experiment 1) to the processing of local displacements in the orientation and proximity domains (Experiment 2) is further evidence of this information-driven processing of visual Gestalts. This differential information-driven processing serves as further support for the notion of Gestalt as predictions proposed here.

These results can also be interpreted in light of other hypotheses beyond RHT and predictive processing models. The global network theory hypotheses^88,89^ and results by Van Vugt et al.^90^ suggest that awareness of visual stimuli is driven by an initial feedforward propagation of stimulus-related information, followed by a nonlinear increase in activity of a frontal-parietal network that will allow that information about stimuli is fed to multiple brain areas through recurrent networks (described in a formal model). According to recent results, weaker stimuli will indeed not cross a certain threshold needed to activate this recurrent fronto-parietal networks. This led the authors to propose that this could explain the mechanism for response omissions and bias in a signal detection theory framework. The different sensitivity to stimuli related to Gestalt-like phenomena around threshold could reveal that the threshold to the engagement of the abovementioned recurrent network would then be lower for emergent features. This would lead to steeper slopes of psychometric functions for Gestalt phenomena, as shown here. But why would this threshold be lower for these phenomena? Another recent formulation related to the global network theory^91^ incorporates Bayesian inference and suggests that different perceptual discrimination task demands are associated to different priors and decision thresholds^92^. The authors, however, do not directly discuss emergent features, as the focus of the model was to assess conscious awareness and stimulus detection more broadly. But with this closing remark, we would like to highlight that multiple alternative narratives that could be evoked to address the issues raised in the current manuscript show remarkable overlaps.

## Conclusion

We have revisited the Gestalts as predictions hypotheses and proposed two complementary experiments as a proof of principle. We degraded stimuli to increase the demands for top-down modulations in order to disambiguate these noisy percepts and assessed the nonlinearities of the psychometric functions as an indicator of the involvement of higher-order visual areas and information-driven processing strategies (as opposed to energy-driven processing). We show that:

(i) stronger degradation of stimuli (associated with increased demands for top-down modulation and use of prior knowledge to disambiguate the noisy sensory input) is related to stronger *Gestaltness* (i.e., larger differences between discrimination of Gestalt and control stimuli);
(ii) the shapes of the psychometric functions derived from Gestalt and control conditions mimic the shape of the CRF derived from extra-striate and striate visual neurons, respectively, and peak differences between Gestalt and control conditions mimic peak differences in the extra-striate and striate responses in Hall et al.^85^, suggesting the differential involvement of higher-order visual areas in Gestalt phenomena and also the differential involvement of information-driven processing mechanisms.

When taken together, these results support the involvement of differential predictive processing mechanisms behind Gestalt phenomena. However, even if one fully bypasses the Gestalts as predictions narrative proposed here, the empirical contribution stands strong. In particular, this work has described how Gestalts behave across the full psychometric function for contrast and other saliency modulations, showing that Gestaltness is maintained even when performance is close to chance and making a case for how Gestalt processing is robust across viewing conditions (and arguably becomes stronger at low visibility conditions). This set of findings significantly broadens our understanding of the generality of visual Gestalt phenomena and complements the current literature regardless of the predictive processing narrative. Future studies should employ this rationale in combination with neuroimaging techniques, allowing for the falsification of some of the predictions made here.

## Data Availability

The datasets generated during and/or analyzed during the current study are available from the corresponding author on reasonable request.
